# Male Infertility and Its Causes in Human

**DOI:** 10.1155/2012/384520

**Published:** 2011-10-20

**Authors:** Toshinobu Miyamoto, Akira Tsujimura, Yasushi Miyagawa, Eitetsu Koh, Mikio Namiki, Kazuo Sengoku

**Affiliations:** ^1^Department of Obstetrics and Gynecology, Asahikawa Medical University, Midorigaokahigashi 2-1-1-1, Asahikawa, Hokkaido 078-8510, Japan; ^2^Department of Urology, Osaka University Graduate School of Medicine, Yamadaoka 2-2, Suita, Osaka 565-0871, Japan; ^3^Department of Integrated Cancer Therapy and Urology, Kanazawa University Graduate School of Medical Science, Takaramachi 13-1, Kanazawa, Ishikawa 920-8641, Japan

## Abstract

Infertility is one of the most serious social problems facing advanced nations. In general, approximate half of all cases of infertility are caused by factors related to the male partner. To date, various treatments have been developed for male infertility and are steadily producing results. However, there is no effective treatment for patients with nonobstructive azoospermia, in which there is an absence of mature sperm in the testes. Although evidence suggests that many patients with male infertility have a genetic predisposition to the condition, the cause has not been elucidated in the vast majority of cases. This paper discusses the environmental factors considered likely to be involved in male infertility and the genes that have been clearly shown to be involved in male infertility in humans, including our recent findings.

## 1. Introduction

One of the most serious social problems facing developed countries today is the declining birth rate, although it is generally not well recognized that the number of infertile couples is on the rise in these countries. While both social (i.e., social progress for women and the resulting increase in the age at which women marry) and environmental (i.e., pollution and global warming) factors are behind part of the increase in the number of patients with infertility, infertility in the male partner contributes to approximately half of all cases.

To date, various techniques, such as *in vitro* fertilization (particularly, intracytoplasmic sperm injection or ICSI) and so-called TESE-ICSI involving the harvesting of sperm from the testes, have been developed for male infertility. Although these methods are steadily producing results, no technique has proven effective for patients with nonobstructive azoospermia, in which there is an absence of mature sperm in the testes. Evidence suggests that many patients with azoospermia have a genetic predisposition to the condition, although the cause has not been elucidated in the vast majority of cases [1]. Conversely, studies using knockout mouse models have recently linked many genes to spermatogenesis, the mechanisms of which are currently being clarified.

These animal findings have yet to be shown applicable to most human cases. This is because identifying the affected genes in humans requires a retrograde genetic approach and because the knockout mouse phenotype is not always faithfully reproduced in humans. This paper discusses the environmental factors considered likely to be involved in male infertility and the genes that have been clearly shown to be involved in male infertility in humans, including our recent research findings.

## 2. Human Male Infertility and Environmental Factors

There has long been debate over whether male reproductive ability is determined by environmental factors, such as those present in the workplace or area of residence. Having a major effect on this debate was the sensational report by Whorton et al. published in 1977, which found that of 25 male workers involved in producing the insecticide dibromo-3-chloropropane (DBCP), 14 were diagnosed as azoospermic or oligospermic [2]. In 1992, Carlsen et al. reported that the previous 50 years saw a marked decrease in sperm count [3]. That same year, Brake and Krause reported that during the period since 1970 in Scotland, sperm counts had decreased by approximately 25% compared with the period prior to 1959, a mean annual rate of 2.1% [4].

Many researchers and clinicians have asserted that societal progress in advanced countries and worsening of the natural environment have likely resulted in decreased male fertility. Long-reported risk factors include working in high temperatures [5], noise associated with manufacturing [6], exposure to radiation [7], electromagnetic waves [8], and a variety of chemical substances [9]. Numerous studies have compared patients with male infertility (oligospermia or azoospermia) to healthy subjects (normal sperm count). To date, proposed risk factors include air temperature [10], automobile driving time per day [11], air pollution [12], regional differences in residential population density [13], mumps [14], stress [15], and alcoholism [16]. On the contrary, many reports indicate the absence of a correlation between environmental factors and male infertility [17, 18]. Thus, there is presently no consistent view on the role of environmental factors and male infertility.

One reason for these discrepancies is that the sample sizes have been insufficient to determine statistically significant differences. The total number of patients with male infertility included in these studies has been small, fewer than 100 patients in nearly all cases. Another reason is that nearly all of these studies have been survey studies using questionnaires, with no objective tests like measuring blood concentrations. Consequently, levels of exposure have been very ambiguous, and selecting healthy men as controls has been problematic.

Many recent studies have included a control group consisting of healthy men selected based on semen findings. However, nearly all of these have been patients who desired to have children and had been examined for infertility on an outpatient basis. Thus, even if the semen findings were normal, it is highly questionable whether the male partner of an infertile couple can be considered healthy. To further elucidate the relationship between environmental factors and male infertility, future studies should incorporate a larger cohort and an appropriate selection of healthy male controls.

## 3. Human Azoospermia and the Y Chromosome

In 1976, Tiepolo and Zuffardi first proposed an explanation for the role of the human Y chromosome in spermatogenesis [19]. They microscopically identified the presence of microdeletions on the long arm of the Y chromosome in six patients with azoospermia and proposed an important spermatogenesis gene in this region. They named this the azoospermia factor (AZF) region. Various subsequent studies have been conducted, particularly by Vogt et al. [20], and in 1995, Reijo et al. examined 89 patients with nonobstructive azoospermia and found that 12 (13%) had a deletion in the AZF region. These results brought recognition to the close relationship between human azoospermia and this region [21]. Vogt et al. further showed the microdeletions to be concentrated in three regions according to the testicular tissue type and divided the AZF region into subregions, AZFa, AZFb, and AZFb [22].

## 4. Azoospermia Culprit Gene Groups

### 4.1. Culprit Gene Identified in the AZF Region

In 1995, Reijo et al. isolated the human deleted-in-azoospermia (DAZ) gene [21]. The human DAZ gene is localized to the AZFc region and encodes 366 amino acids. Its expression is specific to the testis, and the amino acids it encodes have a characteristic structure consisting of an RNP/RRM domain with an RNA binding function and seven tandem repeats of 72 bases [23, 24]. The DAZ gene was the first culprit gene identified as causing human azoospermia as a result of gene mutation. This came 19 years after Tiepolo and Zuffardi first proposed the concept of the AZF region in 1976. This discovery triggered worldwide advances in the search for culprit genes in human azoospermia, and even today this gene is widely known as the most representative of the human azoospermia culprit genes.

Two years later, in 1997, Elliot et al. reported a second azoospermia culprit gene, the RNA-binding motif gene (RBMY, Y chromosome) [25]. As its name suggests, the RBMY gene, like DAZ, encodes an RNA-binding protein; it is located in the AZFb region. The RBMY gene is specifically expressed in germ cells in fetal, adolescent, and adult testes. It is not expressed in somatic cells, such as Sertoli cells. With a deficiency of RBMY, germ cell differentiation is observed only until early meiosis [25]. These research findings strongly suggest that RBMY plays an important role in the process of human spermatogenesis.

In 1999, two years after the report by Elliot et al., Sun et al. identified a new human spermatogenesis gene, USP9Y (DFFRY), in the AZFa region [26]. Using DNA from a total of 576 patients with nonobstructive azoospermia or severe oligospermia (sperm count ≤ 5 million/mL) and 96 healthy subjects, the group performed analysis of single-strand conformation polymorphisms and sequence analysis and identified a 4-bp deletion at the splice-donor site of intron 7 in one patient. Due to this deletion, exon 7 was not expressed at the RNA level, and a frame shift had occurred in this patient, resulting in the loss of approximately 90% of the proteins that would normally have been encoded.

### 4.2. Culprit Gene Identified in Autosomes

The three genes mentioned above are typical spermatogenesis genes localized to the AZF region on the Y chromosome. Many clinicians and researchers have analyzed this region. However, analysis using knockout mice showed numerous autosomal mouse spermatogenesis genes and the importance of meiosis, which is indispensable for the process of spermatogenesis, in the formation of ova. Consequently, we proceeded our analyses based on the hypothesis that culprit genes in human azoospermia are also present in autosomes.

In 2000, Yuan et al. reported a Sycp3 (Scp 3) gene knockout mouse [27]. Synaptonemal complex protein 3 (SYCP3) is a DNA-binding protein related to the synapses involved in the process of germ cell meiosis [28–30]. Both male and female Scycp3 knockout mice develop normally, while the homomutant male has no reproductive potential. The knockout mouse has markedly smaller testis, and histologic analysis indicates meiotic arrest, with a complete absence of round spermatid and elongated spermatid cells, which typically appear after meiosis. Moreover, the mouse has no mature sperm [27]. Compared with wild type, the female Sycp3 gene knockout mouse produces fewer offspring, even though it is capable of gestation and parturition [27]; further analysis has shown the cause to be fetal death *in utero* due to a chromosomal aberration. The frequency of *in utero* fetal death increases with the age of the mouse [31]. Based on these findings in mice, we hypothesized that the SYCP3 gene plays an important role in human spermatogenesis.

To examine our hypothesis, we first established a primer for the site of the human genome sequence homologous to the mouse Sycp3 cDNA at the amino acid level and then used a human testicular cDNA library to isolate human SYCP3 cDNA. The human SYCP3 gene is composed of nine exons and is located on chromosome 12. Its expression is specific to the testis. It encodes 236 amino acids and has two coiled-coil domains [32]. Mutation analysis was performed for all of the coding regions and adjacent introns in 19 patients with azoospermia diagnosed as being caused by a meiotic anomaly, based on histologic analysis. We detected a heterozygous deletion of one adenosine base at the 643-nt site in two of the 19 patients. To rule out polymorphism, we also performed sequence analysis using DNA from 75 healthy men and confirmed no mutations in any of these subjects (*P* = 0.039). Upon repeating detailed histologic analysis, obvious meiotic arrest was observed in the two patients with this mutation. As a result of the loss of germ cells in these patients, a clear decrease in the diameter of the seminiferous tubules and vacuole formation in the testis was seen. Moreover, no round haploid spermatids, elongating spermatids, or mature sperm were seen. These histologic findings are consistent with those for the knockout mouse [27].

A coiled-coil domain present in three regions of the rat Sycp3 gene has been known to play an important role in protein binding [33]. We detected a deletion in this coiled-coil domain. The mutation results in a frame shift, and an early stop codon appears, resulting in an incomplete domain. Consequently, we performed functional analysis of the mutation, by amplifying the sequence associated with the single base deletion at the 643-nt site and normal cDNA using PCR and then inserting them into an expression vector. Following protein extraction, a protein binding assay showed that in patients with the mutation, the SYCP3 gene had lost its protein binding capacity [32]. Based on these findings, we succeeded for the first time in identifying the azoospermia culprit gene SYCP3 located outside the AZF region of the Y chromosome (human chromosome 12).

In 2006, Yatsenko et al. carried out a novel diagnostic strategy using mRNA transcripts from mature sperm in the semen ejaculate [34]. They showed that multiple full-length spermatozoa mRNA that encode candidate infertility-associated proteins can be efficiently screened for mutations by reverse transcriptase-polymerase chain reaction and demonstrated the utility of this approach to diagnose unrecognized genetic defects in severely oligozoospermic men. To test the efficiency of their protocol, they amplified seven genes known to be pre- and/or postmeiotically expressed in germ cells; all were previously characterized using mouse models, and one of them was the kelch-like 10 (*KLHL10*) gene; they previously demonstrated that haploinsufficiency for *Klh10* causes male infertility in mice due to a severe decrease in elongated spermatids and epididymal spermatozoa [35]. The human KLHL10 protein is highly conserved in mammals and consists of 614 amino acids having an N-terminal BTB (bric-a-brac, tramtrack, broad-complex) domain and six tandem C-terminal kelch repeats with an intervening BACK (BTB and C-terminal kelch) domain. The BTB domain of KLHL10 has been suggested to interact with cullin 3 (CUL3) to form a CUL-KLHL10 ubiquitin E3 ligase complex [36] that acts to mediate protein ubiquitination during spermatogenesis. By analyzing KLHL10, they showed the feasibility of an RNA-based approach in diagnosing genetic defects in the germline of infertile men. Their analysis of sperm RNA in 556 oligospermic and 394 normozoospermic individuals revealed seven (1.3%) missense and splicing mutations in this evolutionarily conserved, spermatid-expressed gene. The A313T mutation affects the kelch domain of the protein believed to interact with substrate proteins destined for 26S proteasomal degradation. The Q216P mutation affects the BACK domain thought to orient the substrate in a complex. Since both alleles impair homodimerization of KLHL10 and presumably cause dimer instability, deficiency of protein function may be either due to a dominant-negative effect or due to decreased levels of functional dimers [34].

In 2007, Dieterich et al. reported that homozygous mutation of the human aurora kinase C (*AURKC*) gene yields large-headed polyploidy spermatozoa and causes male infertility in humans [37]. They performed a genome-wide microsatellite scan on ten infertile men presenting a large-headed sperm phenotype. In all of these men, a common region of homozygosity harbors the *AURKC* gene with a single nucleotide deletion in exon 3 (c.144delC) in the *AURKC* coding sequence. This mutation results in premature termination of translation, yielding a truncated protein that lacks the kinase domain. The absence of AURKC causes male infertility owing to the production of large-headed multiflagellar polyploidy spermatozoa. Using *Aurkc* null mice [38], homozygous mice were shown to have no apparent somatic defect and both male and female heterozygous mice to have normal fertility. Moreover, no reduced fecundity was reported in homozygous females, whereas 40% of homozygous males failed to produce pups.

Globozoospermia is a rare (incidence <0.1% in male infertile patients) but severe teratozoospermia, characterized by ejaculates consisting completely of round-headed spermatozoa that lack acrosomes [39]. It originates from a disturbed spermatogenesis, and although the underlying cause is still unknown, a genetic contribution appears to be supported by several familial case reports [40–42] and by three recessive mouse models involving *CSNK2A2*, *HRB*, and *GOPC* [43–45]. Dam et al. investigated an Ashkenazi Jewish family with six brothers (three affected and three healthy) and four sisters [46]. A genome-wide scan analysis of all six brothers was performed using 10 K SNP array. There were approximate 50 known genes in the identified region, chromosome 3q26. The *SPATA16* gene (spermatogenesis-associated 16, also known as *NYD-SP12*) was selected as the most plausible candidate gene, because it is expressed specifically in human testis, as well as primarily in the mouse spermatocytes and spermatids [47]. *SPATA16* is composed of 11 exons encoding a highly conserved protein of 65 kDa (599 aa), which contains a tetratricopeptide repeat (TPR) domain; conservation is very high (92% and 98% in mouse and chimpanzee, resp.) for the TPR domain, a protein-protein interaction domain commonly but exclusively found in cochaperone proteins. Sequence analysis of one of the affected sons revealed a homozygous sequence variation in exon 4 (c.848G→A). The three affected brothers are homozygous, and the two parents and two healthy brothers are heterozygous for the mutation; the third unaffected brother appeared to be homozygous for the wild-type sequence. The mutation is predicted to change an amino acid of a highly conserved residue (p.R283Q) located at the C-terminal end of the highly conserved TPR domain. In addition, the c.848G→A mutation affects the last nucleotide of exon 4 and disrupts the 5 ′ splice site of intron 4. Thus, the c.848G→A mutation leads to inappropriate splicing of exon 4 that causes disruption of the TPR domain.

## 5. Male Infertility and Genetic Polymorphism

Genetic polymorphisms may also increase susceptibility to some forms of male infertility. We have identified polymorphisms of several genes that are associated with the human azoospermic population—*MEI1, PRDM9 (MEISETZ), SPATA17, PARP-2*, and *UBR2* genes are genetic risk factors for the patients with azoospermia by meiotic arrest [48–52], and polymorphisms of the *SEPTIN12* gene are associated with patients with Sertoli cell-only syndrome [53]. Genetic polymorphisms and male infertility have been under much investigation recently. Some genes identified to be associated with male infertility in the past three years include: *MTHFR, SHBG, Piwi, CYP19A1, NER, GSTM1, BCL2, ESR1, ESR2, eNOS, TNP1, SOHLH1, EPPIN, GSTT1, TSSK6, TSSK2, MDR1, MSH5, MLH3, H2BFWT, PACRG*, and *FASLG* [54–74]. Despite identification of these genes, neither the mechanisms of human spermatogenesis nor the association of these genes with each other is well known. We believe that environment is an important factor associated with genetic polymorphisms in human spermatogenesis. Further analysis is thus strongly needed to determine the association between genetic polymorphisms and environmental factors.

## 6. Conclusion

This paper discussed the gene groups that have been reported to date to play a role in human spermatogenesis, as well as our recent research findings. Recent mouse studies and our genetic polymorphism studies suggest that numerous human spermatogenesis gene groups are present in regions other than the AZF region of the Y chromosome, for which analysis has progressed worldwide. As patients with a microdeletion in the AZF region of the Y chromosome account for approximately 13% of those with human azoospermia (recent reports indicate an even lower percent of approximately 7%), new human azoospermia culprit genes are expected to be identified in areas other than the Y chromosome.

Although striking progress has been achieved in recent years in elucidating the mechanism of spermatogenesis using knockout mice, few studies have applied these findings to humans. A reason for this is the limitation of reproducing the knockout mouse phenotype faithfully in humans.

When couples with a desire to have children visit our hospital (Asahikawa Medical College Hospital) to examine for infertility and the man is diagnosed as azoospermic based on a semen test, a urologist will perform further examinations such as endocrinology tests of the man, testicular ultrasound, and chromosome tests (particularly, determination of the presence of microdeletions in the AZF region of the Y chromosome). Although the presence of sperm in the testes can to some extent be determined based on the results of these tests, a true determination can only be made when testicular sperm extraction is performed. Further research on human spermatogenesis is necessary to establish a diagnostic method that is less invasive and that reduces the mental, physical, and financial burden on the patient.
